# SAAED: Embedding and Deep Learning Enhance Accurate Prediction of Association Between circRNA and Disease

**DOI:** 10.3389/fgene.2022.832244

**Published:** 2022-02-22

**Authors:** Qingyu Liu, Junjie Yu, Yanning Cai, Guishan Zhang, Xianhua Dai

**Affiliations:** ^1^ School of Electronics and Information Technology, Sun Yat-Sen University, Guangzhou, China; ^2^ Macquarie Business School, Macquarie University, Sydney, NSW, Australia; ^3^ College of Information Science and Technology, Jinan University, Guangzhou, China; ^4^ College of Engineering, Shantou University, Shantou, China

**Keywords:** circRNA–disease association, embedding, neural network, Pseudo-Siamese network, deep learning

## Abstract

Emerging evidence indicates that circRNA can regulate various diseases. However, the mechanisms of circRNA in these diseases have not been fully understood. Therefore, detecting potential circRNA–disease associations has far-reaching significance for pathological development and treatment of these diseases. In recent years, deep learning models are used in association analysis of circRNA–disease, but a lack of circRNA–disease association data limits further improvement. Therefore, there is an urgent need to mine more semantic information from data. In this paper, we propose a novel method called Semantic Association Analysis by Embedding and Deep learning (SAAED), which consists of two parts, a neural network embedding model called Entity Relation Network (ERN) and a Pseudo-Siamese network (PSN) for analysis. ERN can fuse multiple sources of data and express the information with low-dimensional embedding vectors. PSN can extract the feature between circRNA and disease for the association analysis. CircRNA–disease, circRNA–miRNA, disease–gene, disease–miRNA, disease–lncRNA, and disease–drug association information are used in this paper. More association data can be introduced for analysis without restriction. Based on the CircR2Disease benchmark dataset for evaluation, a fivefold cross-validation experiment showed an AUC of 98.92%, an accuracy of 95.39%, and a sensitivity of 93.06%. Compared with other state-of-the-art models, SAAED achieves the best overall performance. SAAED can expand the expression of the biological related information and is an efficient method for predicting potential circRNA–disease association.

## 1 Introduction

CircRNA is a non-coding RNA formed by reverse splicing (Nigro et al., 1991; Danan et al., 2012; Salzman et al., 2013) and performs multiple functions in the nucleus, cytoplasm, and extracellular matrix (Li et al., 2018). In the nucleus, circRNA can regulate the splicing of their linear mRNA counterpart (Ashwal-Fluss et al., 2014; Zhang et al., 2014; Kelly et al., 2015) and control the transcription of parental genes (Li et al., 2015). In the cytoplasm, as miRNA sponges (Hansen et al., 2013) and ceRNAs (Salmena et al., 2011), circRNAs competitively bind with miRNA, which can interact with target mRNAs to induce mRNA degradation and translational repression (Fabian and Sonenberg, 2012). Moreover, it plays a regulatory role through binding proteins (Memczak et al., 2013), and can be translated (Chen and Sarnow, 1995; Conn et al., 2015; Wang and Wang, 2015). *In vitro*, circRNA can serve as an ideal biomarker, because it is more stable compared to other linear non-coding RNA molecules (Zhang and Xin, 2018; Shang et al., 2019; Slack and Chinnaiyan, 2019).

As described above, circRNA engages in a large number of biological processes and is associated with various diseases. It has been found that N^6^-methyladenosine-modified CircRNA-SORE, sequestering miR-103a-2-5p and miR-660-3p by acting as a microRNA sponge, sustains sorafenib resistance in hepatocellular carcinoma by regulating *β*-catenin signaling (Xu et al., 2020). In addition, it has been proved that circMRPS35 governs histone modification in anticancer treatment and advocates for triggering the circMRPS35/KAT7/FOXO1/3a pathway to combat gastric cancer (Jie et al., 2020).

So, analyzing the relationship between circRNA and disease can help understand the disease mechanism, treatments, and diagnoses (Ghosal et al., 2013; Liu et al., 2019a). However, traditional experiments are time-consuming and a lack of circRNA–disease association data limits further improvement. In recent years, various models are developed for association analysis. These models could be divided into three categories. The first model category involves the use of Gaussian Interaction Profile (GIP) or JACCARD index to calculate the similarity between circRNA and between diseases, and then the application of different models to extract features from the similarity matrix for further analysis, such as the KATZ measure (Fan et al., 2018a), path weighting methods (Lei et al., 2018), and k-nearest neighbor method with decreasing weight (Yan et al., 2018).

The second category is based on machine learning and deep learning. Wang et al. (2020a) propose an efficient computational method based on multi-source information combined with deep convolutional neural network (CNN) to predict circRNA–disease associations. The method extracts the hidden deep feature through the CNN and finally sends them to learning machine classifier for prediction. GCNCDA (Wang et al., 2020b) is based on the Fast learning with Graph Convolutional Networks (FastGCN) algorithm to predict the potential disease-associated circRNA. Specifically, the method first forms the unified descriptor by fusing disease semantic similarity information, disease, and circRNA GIP kernel similarity information. They use traditional methods to deal with association, which is difficult to integrate more knowledge.

The third category is based on embedding. In deep learning, the vector transformed by the embedding model is called embedding vector (Mikolov et al., 2013a). Recently, large-scale pre-trained models are the most popular embedding models. They can effectively introduce large amount of information into the embedding vectors with self-supervised learning and unlabeled data. Word2Vec (Mikolov et al., 2013b) and BERT (Jacob et al., 2019) are famous embedding models in natural language processing to calculate the embedding vector. Bordes et al. (2011) propose a structured distributed embedding method to learn the entities relations in Knowledge Bases. The embedding space allows to estimate the probability density of any relation between entities, preserves the knowledge of the original data, and presents the interesting ability of generalizing to new reasonable relations. In the field of bioinformatics, codon-based encoding (Zhang et al., 2019) and rna2vec (Xiao et al., 2018) are two embedding models transforming the codon and nucleobase into embedding vector. However, nucleobase and codon are heavily recurring in RNA sequences and require complex models to extract their semantics information. Xiao et al. (2021) propose an embedding model to calculate the embedding vector of circRNA and disease, but it cannot fuse more new association information into the embedding vector, and does not use large-scale learning methods such as deep learning.

Therefore, we proposed a novel neural network embedding model called Entity Relation Network (ERN) to calculate the embedding vector of diseases and circRNA. The model introduces various entity association information, i.e., circRNA–miRNA, circRNA–disease, disease–gene, disease–lncRNA, disease–drug, and disease–miRNA association, so the embedding vector contains more information than the previous model for extraction and analysis. Compared with traditional embedding vector, ERN can generate a fixed low-dimensional embedding vector, which is learnable and can reduce computational complexity. This means similar circRNAs or diseases will approach each other in the embedding space during the training process, hence making the model easier to converge and analyze the associations. By using the embedding vectors calculated by ERN, we have made significant progress in circRNA–disease association analysis.

## 2 Materials and Methods

### 2.1 Dataset of circRNA Association

In this study, we implement the model on the CircR2Disease (Fan et al., 2018b) and Circbank (Liu et al., 2019b) to calculate the embedding of the circRNA. CircR2Disease database supplies experimentally varied circRNA–disease associations, which can be freely obtained from http://bioinfo.snnu.edu.cn/CircR2Disease/. Currently, CircR2Disease has collected 725 associations between 661 circRNAs and 100 diseases from existing literatures. CircR2Disease is the benchmark dataset for evaluation of the circRNA–disease association analysis. Circbank is a comprehensive database of human circRNA with 16,844,375 circRNA–miRNA predicted associations between 1,917 miRNAs and 140,790 circRNAs, which can be freely obtained from http://www.circBANK.cn.

### 2.2 Dataset of Disease Association

We integrate DisGeNET (Piñero et al., 2016), HMDD (Huang et al., 2019), LncRNADisease (Bao et al., 2019), and Comparative Toxicogenomics Database (CTD) (Davis et al., 2021) for the calculation of the embedding of disease. DisGeNET is a dataset of disease–gene association, which can be freely obtained from https://www.disgenet.org/. It contains 1,134,942 gene-disease associations, between 21,671 genes and 30,170 diseases. HMDD is a dataset of disease–miRNA with 35,547 miRNA–disease associations between 1,206 miRNAs and 893 diseases, which can be freely obtained from https://www.cuilab.cn/hmdd. LncRNADisease is a dataset with 20,595 LncRNA–disease associations and 1,004 circRNA–disease associations from 19,166 lncRNAs, 823 circRNAs, and 529 diseases, which can be freely obtained from http://www.rnanut.net/lncrnadisease/. CTD is a dataset with 224,627 disease–drug associations from 10,152 drugs and 3,278 diseases, which can be freely obtained from http://ctdbase.org/.

Table1 shows the number of different types of associations in SAAED. We use two adjacency matrices to represent the associations between circRNA, disease, and other biological entities, respectively. When the specific circRNA or disease and specific entity is associated, the element is assigned a value of 1, otherwise 0.

**TABLE 1:** The number of different types of associations in SAAED.

Dataset	Association	Amount of relation	Amount of Entity 1	Amount of Entity 2
CircR2Disease	CircRNA–Disease	725	661	100
Circbank	CircRNA–miRNA	16,844,375	140,790	1,917
DisGeNET	Disease–Gene	1,134,942	30,170	21,671
HMDD	Disease–miRNA	35,547	893	1,206
LncRNADisease	Disease–LncRNA	20,595	529	19,166
CTD	Disease–Drug	224,627	3,278	11,152
Total	—	18,260,811	—	—

### 2.3 Method Overview

**FIGURE 1:**
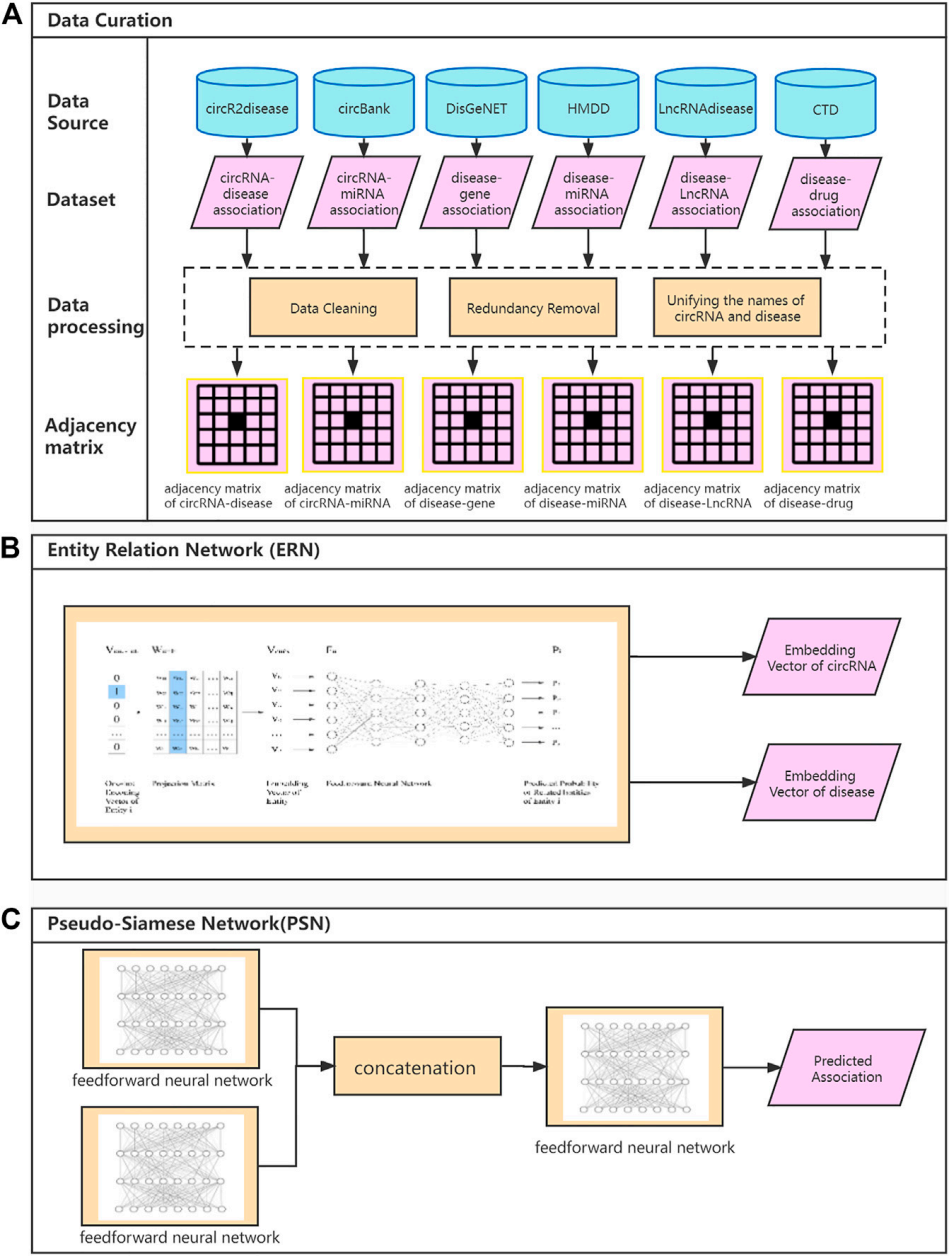
SAAED flowchart. **(A)** An outline of flowchart of data curation. (i) The association information is extracted from data sources. (ii) Data preprocess deal with data cleaning, redundancy removal, and unifying the names of circRNA and diseases from different datasets. (iii) The association information, i.e., represented by the adjacency matrix. **(B)** The Entity Relation Network (ERN) calculates the embedding vectors. **(C)** The Pseudo-Siamese Network calculates the probability of association with the embedding vector of circRNA and disease.

SAAED consists of two parts, which is shown in Figure 1. The first part is the ERN for embedding circRNA and diseases. The second one is the Pseudo-Siamese network (Koch et al., 2015), which is used to analyze the probability of association between circRNA and diseases. More specifically, the input to SAAED is entity association information, which can be represented by an adjacency matrix. The size of the adjacency matrix is arbitrary. Therefore, the model can easily introduce information about semantic entities.

### 2.4 Embedding and Entity Relation Network

The similarity between circRNA or diseases can be analyzed through their structure or function. Structure refers to sequence information or spatial structure of the circRNA and disease. Function refers to the interaction between circRNA, disease, and other biological entities. Researchers often use one-hot encoding vector to convert these information into embedding vectors and analyze them by various models (Fan et al., 2018a; Lei et al., 2018; Yan et al., 2018; Wang et al., 2020a; Wang et al., 2020b), but one-hot encoding increases the computational complexity as the information used increases. In addition, the matrix of the one-hot encoding is sparse, and the computational efficiency is low. Hence, we try to construct a deep learning model called Entity Relation Network (ERN) to learn a fixed-length continuous embedding vector from the associated information.

In ERN, we construct the embedding model to transform a large amount of association information, such as circRNA–disease, circRNA–miRNA, disease–gene, disease–miRNA, disease–lncRNA, and disease–drug associations, into embedding vectors so that the model can analyze heterogeneous information simultaneously. In terms of solving biological association problems, embedding of ERN has the following advantages:

1) Embedding vector has strong expression ability. Fixed length embedding vector learned by ERN is used to introduce semantic knowledge without increasing computational complexity, so as to solve the flexibility of semantic expansion.
2) Embedding expression is more accurate. ERN translates the 0,1 matrix into a fixed length embedding vector by neural network learning algorithm, and optimizes the correlation degree by automatic learning, which is more accurate than the Euclidean distance correlation measure of GIP.

ERN adopts a probabilistic feedforward neural network language model (Bengio et al., 2003; Koch et al., 2015) to extract information from association data and further transforms it into an embedding vector. The association data between entity A and entity B can be represented by a adjacency matrix M, where I represents the number of entity A, and J represents the number of entity B. When entity A(i) is associated with entity B(j), the element M(A(i), B(j)) of matrix M is assigned the value of 1. Otherwise, it has a value of 0.

$$\begin{array}{c} M\left(A\left(i\right),B\left(j\right)\right)\{\begin{array}{c} 1,\;\;if\;A\left(i\right)\;\text{is}\;\mathrm{associated}\;\mathrm{with}\;B\left(j\right)\;\\ 0,\;\mathrm{otherwise}\; \end{array} \end{array}$$ (1)

**FIGURE 2:**
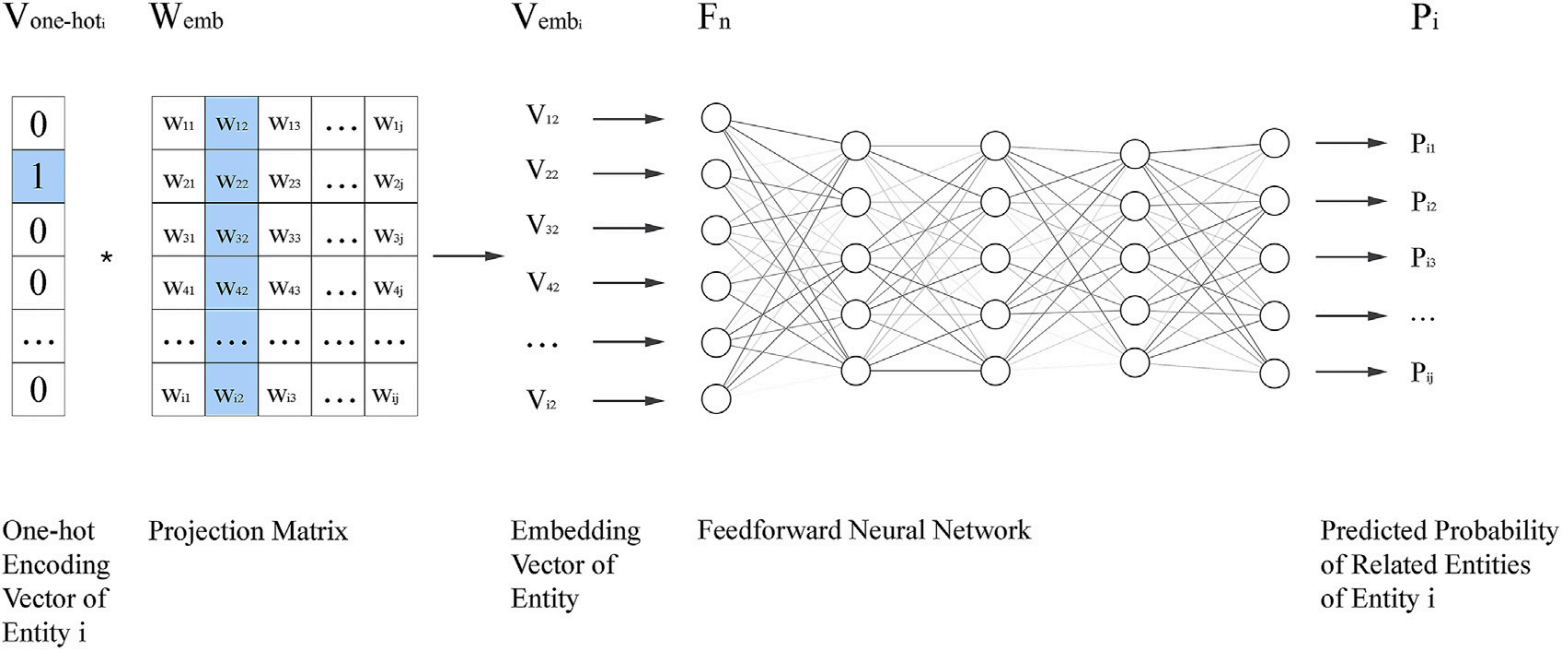
The flowchart of the Entity Relation Network (ERN). $V_{\text{one}-{\text{hot}}_{i}}$ is the one-hot encoding vector of the entity A(i), $W_{\text{emb}}$ is the projection matrix, $F_{n}$ is a feedforward neural network, and $P_{i}$ is the predicted probability vector of A(i), and $V_{\text{emb}}$ is embedding vector of entity A(i). One-hot encoding vector $V_{\text{one}-{\text{hot}}_{i}}$ selects the specific column of parameter matrix $W_{\text{emb}}$ as the embedding vector $V_{\text{emb}}$ of entity i, which is used for predicting the related entities. After training the embedding vector, i.e., the parameter matrix is learned.

The adjacency matrix is used to represent entity association information. ERN projects the adjacency matrix onto the embedding vector, which consists of an input layer, a projection layer, a feedforward neural network, and an output layer. The flowchart of ERN is shown in Figure 2.

We define the vector V(A(i)) to represent all associated entities of A(i), which is the *i*th row of the adjacency matrix M. ERN can be trained to predict probability of each associated entity related to A(i), and the feedforward neural network is used to analyze the embedding vector and output the probability.

$$\begin{array}{c} V_{{\mathrm{emb}}_{i}}=V_{\mathrm{one}-{\mathrm{hot}}_{i}}*W_{\mathrm{emb}} \end{array}$$ (2)

$$\begin{array}{c} P_{i}=F_{n}\left(V_{{\mathrm{emb}}_{i}}\right) \end{array}$$ (3)

Where $V_{\mathrm{one}-{\mathrm{hot}}_{i}}$ is the one-hot encoding vector of the entity A(i), $W_{\mathrm{emb}}$ is the projection matrix, $F_{n}$ is a feedforward neural network, and $P_{i}$ is the predicted probability vector of A(i), and $V_{{\mathrm{emb}}_{i}}$ is the embedding vector of entity A(i).

The ERN training uses one-hot encoding vector and adjacency matrix as the input and probability of association as the output. Taking the adjacency matrix as the learning goal, the ERN uses the linear transformation to generate the embedding vector by deep leaning algorithm.

The loss function of ERN is the mean square error,

$$\begin{array}{c} \mathrm{Loss}=\overset{I}{\underset{i=1}{\sum }}\sum_{j=1}^{J}{\left(P_{i_{j}}-\text{V}{\left(\text{A}\left(\text{i}\right)\right)}_{j}\right)}^{2} \end{array}$$ (4)

where $P_{i_{j}}$ is the *j*th element of $P_{i}$ , which is the probability that A(i) is associated with B(j); $\text{V}{\left(\text{A}\left(\text{i}\right)\right)}_{j}$ is the *j*th element of V(A(i)) , which indicates whether A(i) is associated with entity B(j).

GIP is the most commonly used encoding method in the past. Compared with GIP, ERN has three advantages. Firstly, ERN can introduce any amount of external information into the embedding vectors. Secondly, the size of the embedding vector is fixed regardless of the number of entities and information introduced, keeping the complexity of the model constant. Thirdly, by reducing the loss function during training, the representation of features is enhanced, especially for two entities with high similarity, and their embedding vectors will be closer in the embedding space. It should be noted that in the training of ERN, we should overfit the model so that the embedding vector calculated by ERN can accurately reflect the relationship, i.e., the distance between different entities.

The use of embedding vectors with significant features makes the Pseudo-Siamese network easier to converge and analyze, so the overall model achieves better performance than previous models.

### 2.5 Calculation of the Disease Embedding Vector

Based on the disease–gene, disease–miRNA, disease–lncRNA, and disease–drug association data, the disease related adjacency matrix D is constructed:

$$\begin{array}{c} D\left(d\left(i\right),e\left(j\right)\right)=\{\begin{array}{c} 1,\;\;\mathrm{if}\;\mathrm{diseased}\left(i\right)\;\mathrm{is}\;\mathrm{associated}\;\mathrm{with}\;\mathrm{relevant}\;\mathrm{entity}\;e\left(j\right)\\ 0,\;\mathrm{otherwise}\;\;\;\;\;\;\;\;\;\;\;\;\;\;\;\;\;\;\;\;\;\;\; \end{array} \end{array}$$ (5)

V(d(i)) is the *i*th row of the adjacency matrix D, which reflects the associated genes of disease d(i) .

The input of the model is the one-hot encoding vector of disease d(i) .The details of the model are as follows:

$$\begin{array}{c} VD_{{\mathrm{emb}}_{i}}=VD_{\mathrm{one}-{\mathrm{hot}}_{i}}*WD_{\mathrm{emb}} \end{array}$$ (6)

$$\begin{array}{c} PD_{i}=\mathrm{Sigmoid}\left(\mathrm{Sigmoid}\left(ReLU\left(VD_{{\mathrm{emb}}_{i}}\right)*W_{1}\right)*W_{2}\right) \end{array}$$ (7)

$$\begin{array}{c} \mathrm{Loss}=\overset{I}{\underset{i=1}{\sum }}\sum_{j=1}^{J}{\left(PD_{\;i}-V\left(d\left(i\right)\right)\right)}^{2} \end{array}$$ (8)

Where ${\text{VD}}_{\mathrm{one}-{\mathrm{hot}}_{\text{i}}}$ is the one-hot encoding vector of disease d(i) , ${\text{WD}}_{\mathrm{emb}}$ is the projection matrix, ${\text{VD}}_{{\mathrm{emb}}_{\text{i}}}$ is the embedding vector of disease d(i) , $W_{1}$ and $W_{2}$ are the weights of feedforward neural network, and $PD_{\;i}$ is the predicted probability indicating which gene may be associated with the diseased(i) .

### 2.6 Calculation of circRNA Embedding Vector

Based on the circRNA–disease and circRNA–miRNA association data, the circRNA-related adjacency matrix C is constructed, and the embedding vector of circRNA is calculated.

$$\begin{array}{c} \begin{array}{c} C\left(c\left(i\right),e\left(j\right)\right)=\{\begin{array}{c} 1,\;\mathrm{if}\;\mathrm{circRNAc}\left(i\right)\;\mathrm{is}\;\mathrm{associated}\;\mathrm{with}\;\mathrm{relevant}\;\mathrm{entity}\;e\left(j\right)\\ 0,\;\mathrm{otherwise} \end{array}\; \end{array} \end{array}$$ (9)

$$\begin{array}{c} \text{Related}\;\text{diseases}\;\text{of}\;{\text{circRNA}}_{\text{i}}=V\left(c\left(i\right)\right) \end{array}$$ (10)

$$\begin{array}{c} VC_{{\mathrm{emb}}_{i}}=VC_{\mathrm{one}-{\mathrm{hot}}_{i}}*WC_{\mathrm{emb}} \end{array}$$ (11)

$$\begin{array}{c} PC_{i}=\text{Sigmoid}\left(\text{Sigmoid}\left(ReLU\left(VC_{{\mathrm{emb}}_{i}}\right)*W_{1}\prime \right)*W_{2}\prime \right) \end{array}$$ (12)

$$\begin{array}{c} \text{Loss}=\overset{I}{\underset{i=1}{\sum }}\sum_{j=1}^{J}{\left(PC_{i}-V\left(c\left(i\right)\right)\right)}^{2} \end{array}$$ (13)

Where $VC_{\mathrm{one}-{\mathrm{hot}}_{i}}$ is the one-hot encoding vector of circRNA  c(i) , $WC_{\mathrm{emb}}$ is the projection matrix, ${\text{VC}}_{{\mathrm{emb}}_{\text{i}}}$ is the embedding vector of circRNA c(i) , $W_{1}\prime$ and $W_{2}\prime$ are the weights of the model, and $PC_{i}$ is the predicted probability indicating which disease may be associated with circRNA c(i) .

### 2.7 Information Fusion and circRNA–Disease Association Analysis

We used circRNA–disease association data from CircR2Disease as positive samples and randomly selected the same number of associations as negative samples. Although unconfirmed circRNA–disease associations may be regarded as negative samples, the probability is significantly lower.

**FIGURE 3:**
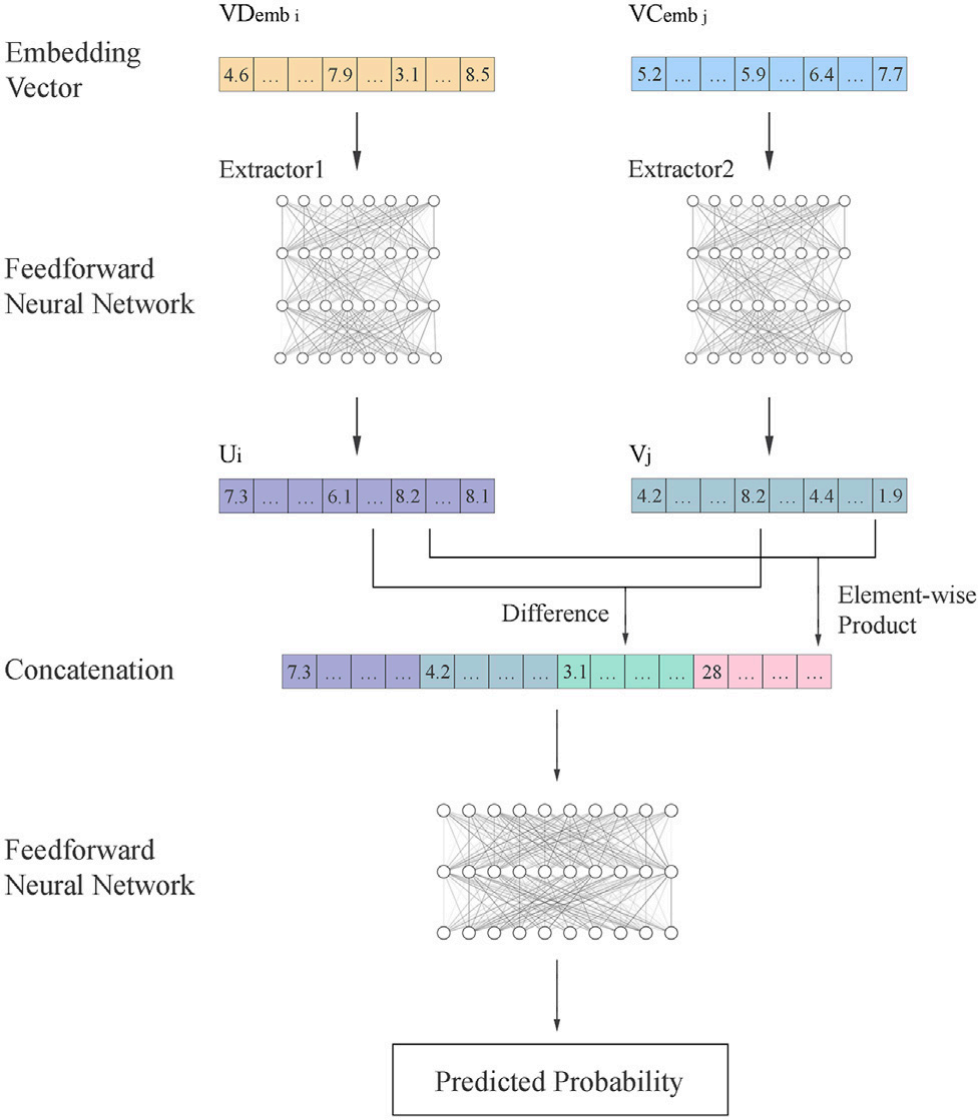
Flowchart of Pseudo-Siamese network. Two feedforward neural networks are used as the extractor to extract features u and v from the embedding vector ${\text{VD}}_{\text{emb}}$ and ${\text{VC}}_{\text{emb}}$ . The difference and element-wise product of u and v are concatenated with u and v for analyzing association by a Feedforward neural network.

The Pseudo-Siamese network is adopted to fuse information from circRNA and diseases to infer their relationship. The flowchart is shown in Figure 3. The embedding vectors of circRNA and disease are calculated based on different information. The Pseudo-Siamese network can learn two different transformations to project the embedding vectors of circRNA and diseases from the original semantic space into a new semantic space for analysis.

The Pseudo-Siamese network has two inputs. After feature extraction, the features are concatenated and analyzed by feedforward neural network to output the probability of association. $VD_{\mathrm{emb}}$ and $VC_{\mathrm{emb}}$ are the embedding vectors calculated by ERN. Two different feedforward neural networks as the extractors extract feature vectors from these two inputs, respectively:

$$\begin{array}{c} u={\mathrm{Extractor}}_{1}\left(VD_{\mathrm{emb}}\right) \end{array}$$ (14)

$$\begin{array}{c} v={\mathrm{Extractor}}_{2}\left(VC_{\mathrm{emb}}\right) \end{array}$$ (15)

Where *u* and *v* are the feature vectors transformed from the embedding vectors of circRNA and disease. The difference and element-wise product of *u* and *v* are calculated to enhance the inference of local information, and then concatenated with *u* and *v*. Finally, another feedforward neural network is used to calculate the probability.

$$\begin{array}{c} P=\mathrm{Sigmoid}\left(F_{n}\left(\left[u;v;u-v;u⊙v\right]\right)\right) \end{array}$$ (16)

Where *P* is the predicted probability, $F_{n}$ is the feedforward neural network. The Sigmoid function is used to limit the predicted probability to a range from 0 to 1.

### 2.8 General Entity Embedding

After training the ERN and obtaining the embedding vector, the embedding vector can be used as the input to the feedforward neural network in ERN,

$$\begin{array}{c} P_{i}=F_{n}\left(V_{{\mathrm{emb}}_{i}}\right) \end{array}$$ (17)

If we derive the inverse function of $F_{n}$ and use V(A(i)) as an input to $F_{n}^{-1}$ , we have

$$\begin{array}{c} V_{{\mathrm{emb}}_{i}}=F_{n}^{-1}\left(\text{V}\left(\text{A}\left(\text{i}\right)\right)\right) \end{array}$$ (18)

Where $F_{n}^{-1}$ is the inverse function of $F_{n}$ .

Since it is difficult to derive the inverse function, a new feedforward neural network can be used to fit the inverse function $F_{n}^{-1}$ with V(A(i)) and $V_{{\mathrm{emb}}_{i}}$

$$\begin{array}{c} V_{{\mathrm{emb}}_{i}}=F_{m}\left(\text{V}\left(\text{A}\left(\text{i}\right)\right)\right) \end{array}$$ (19)

Where $F_{m}$ is a feedforward neural network.

The above function indicates that the embedding vector can be calculated directly by using the association information, regardless of whether the disease is in CircR2Disease or not. Thus, the scope of circRNA–disease association analysis is greatly expanded. $F_{m}$ can be regarded as an embedding function learned by ERN from association information. Unlike GIP or other formulas, $F_{m}$ can be adjusted based on the data, so as to introduce more information into the embedding vector and improve the quality of the embedding vector.

## 3 Results

### 3.1 Performance Metrics

To evaluate the performance of SAAED, we used the fivefold cross-validation to divide the data into training sets and testing sets in the ratio of 4:1, i.e., 1,000 data are used for training and 250 data are used for testing. The fivefold cross-validation can make full use of the data to train and test the generalization capability of the model, and avoid the adverse effects of unreasonable division of the training and testing sets on model evaluation. The model is evaluated by accuracy (Accu.), sensitivity (Sen.), precision (Prec.), F1 score (F1), and AUC. They are defined as:

$$\begin{array}{c} \mathrm{Accu}.=\frac{TP+TN}{TP+TN+FP+FN} \end{array}$$ (20)

$$\begin{array}{c} \mathrm{Sen}\text{.}=\frac{TP}{TP+FN} \end{array}$$ (21)

$$\begin{array}{c} \mathrm{Prec}\text{.}=\frac{TP}{TP+FP} \end{array}$$ (22)

$$\begin{array}{c} F1=\frac{2TP}{2TP+FP+FN} \end{array}$$ (23)

where TP, FP, TN, and FN represent the number of true positive, false positive, true negative, and false negative, respectively. TP is the number of positive (given circRNA is related with given disease) correctly classified by the model; FP is the number of negative (given circRNA is not related with given disease) misclassification; TN is the number of negative (unrelated) correctly classified by the model; FN is the number of positive that is wrongly labeled.

### 3.2 Model Performance Evaluation

SAAED is implemented on the circR2Disease dataset to evaluate its ability to predict potential circRNA–disease associations. The results of fivefold CV are summarized in Table 2.

**TABLE 2:** Result of fivefold CV generated by SAAED on the CircR2Disease Dataset.

Test set	Accu.(%)	Sen.(%)	Prec.(%)	F1(%)	AUC(%)
1	95.59	93.33	99.41	96.28	99.08
2	94.08	92.57	95.86	94.19	98.47
3	95.56	93.26	98.22	95.68	99.26
4	96.15	93.82	98.82	96.25	98.36
5	95.56	92.31	99.41	95.73	99.44
Average	95.39±0.77	93.06±0.61	98.34±1.47	95.63±0.85	98.92±0.48

According to statistical indicators, the average accuracy of the model is 95.39%, the average sensitivity is 93.06%, the average precision is 98.34%, the average F1 score is 95.63%, and the AUC is 0.9892, with all standard deviations less than 2. This indicated that SAAED achieved excellent robustness in the CircR2Disease dataset and is able to effectively predict circRNA–disease associations.

**FIGURE 4:**
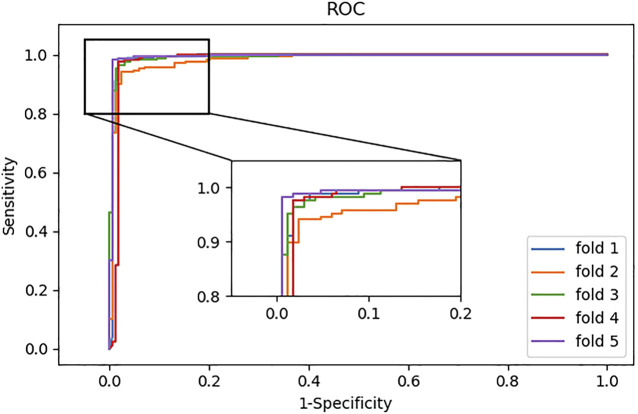
ROC curves of fivefold CV obtained by SAAED on the CircR2Disease Dataset.

In addition, we also plotted the ROC curves generated by the model. As shown in Figure 4, the ROC curves can reach the upper left corner of the graph.

We made a comparison of KATZHCDA (Liu et al., 2019a), PWCDA (Ghosal et al., 2013), DWNN-RLS (Fan et al., 2018a), and GCNCDA (Yan et al., 2018). The results are shown in Table 3. According to the fivefold CV AUC scores, SAAED obtained the highest AUC.

**TABLE 3:** The fivefold CV AUC scores generated by various models on the same benchmark dataset CircR2Disease.

Methods	SAAED	GCNCDA	DWNN-RLS	PWCDA	KATZHCDA
AUC(%)	98.92	90.90	88.54	89.00	79.36

### 3.3 Cases Studies of the Association Between circRNA and Breast Cancer/HCC

In order to evaluate the practical value of SAAED, we choose the model with highest AUC to make predictions for circRNA associated with breast cancer and HCC, two diseases for which sufficient data are available in the CircR2Disease dataset to avoid model bias due to a lack of data as much as possible. We used the embedding vector of circRNA, breast cancer, and HCC as inputs to the model, and the predicted probability can reflect the relationship between the specified circRNA and disease.

As shown in Table 4, 16 of the 20 data with the highest predicted probabilities are confirmed to be associated with breast cancer, and according to the literature, 6 prediction results (hsa_circ_0000615, CDR1as, ciRS-7, cZNF609, hsa_circ_0007386, and circSMARCA5) are newly identified by the model.

**TABLE 4:** The top 20 breast cancer-related candidate circRNA.

Rank	circRNA	Evidence (PMID)	Rank	circRNA	Evidence (PMID)
1	hsa_circ_0000615	32398664	11	hsa_circ_0001445	Unconfirmed
2	CDR1as	31245927	12	circSMARCA5	32838810
3	iRS-7	30072582	13	hsa_circ_0001785	CircR2Disease
4	cZNF609	32398664	14	hsa_circ_0011946	CircR2Disease
5	hsa_circ_0007386	32808350	15	hsa_circ_0008717	CircR2Disease
6	circHIPK3	CircR2Disease	16	hsa_circ_0000732	CircR2Disease
7	hsa_circ_0000284	CircR2Disease	17	circRNA-001283	CircR2Disease
8	mmu_circ_0001878	Unconfirmed	18	hsa_circ_0001721	CircR2Disease
9	hsa_circ_0067934	Unconfirmed	19	circABCB10	CircR2Disease
10	hsa_circ_0004712	Unconfirmed	20	hsa_circ_0086241	CircR2Disease

As shown in Table 5, 13 of the 20 data with the highest predicted probabilities are confirmed to be associated, and according to the literature, 4 of the associated circRNA (hsa_circ_0000615, cZNF609, has_circ_0000520, and hsa_circ_0000517) are newly identified by the model.

**TABLE 5:** The top 20 hepatocellular carcinoma-related candidate circRNA.

Rank	circRNA	Evidence (PMID)	Rank	circRNA	Evidence (PMID)
1	hsa_circ_0000615	32398664	11	hsa_circ_0001819	Unconfirmed
2	CDR1as	CircR2Disease	12	hsa_circRNA_000598	Unconfirmed
3	ciRS-7	CircR2Disease	13	hsa_circ_0000520	27258521
4	cZNF609	32398664	14	hsa_circ_0004018	CircR2Disease
5	hsa_circ_0007386	Unconfirmed	15	hsa_circ_0005986	CircR2Disease
6	circHIPK3	CircR2Disease	16	circRNA_000839	CircR2Disease
7	hsa_circ_0000284	CircR2Disease	17	hsa_circ_0056731	Unconfirmed
8	mmu_circ_0001878	Unconfirmed	18	hsa_circ_0001400	Unconfirmed
9	hsa_circ_0067934	CircR2Disease	19	hsa_circ_0067531	CircR2Disease
10	hsa_circ_0004712	Unconfirmed	20	hsa_circ_0000517	31750237

In addition, recall rates are analyzed for data with probabilities greater than 0.9, with a recall rate of 0.9310 for breast cancer and 0.7647 for HCC.

**FIGURE 5:**
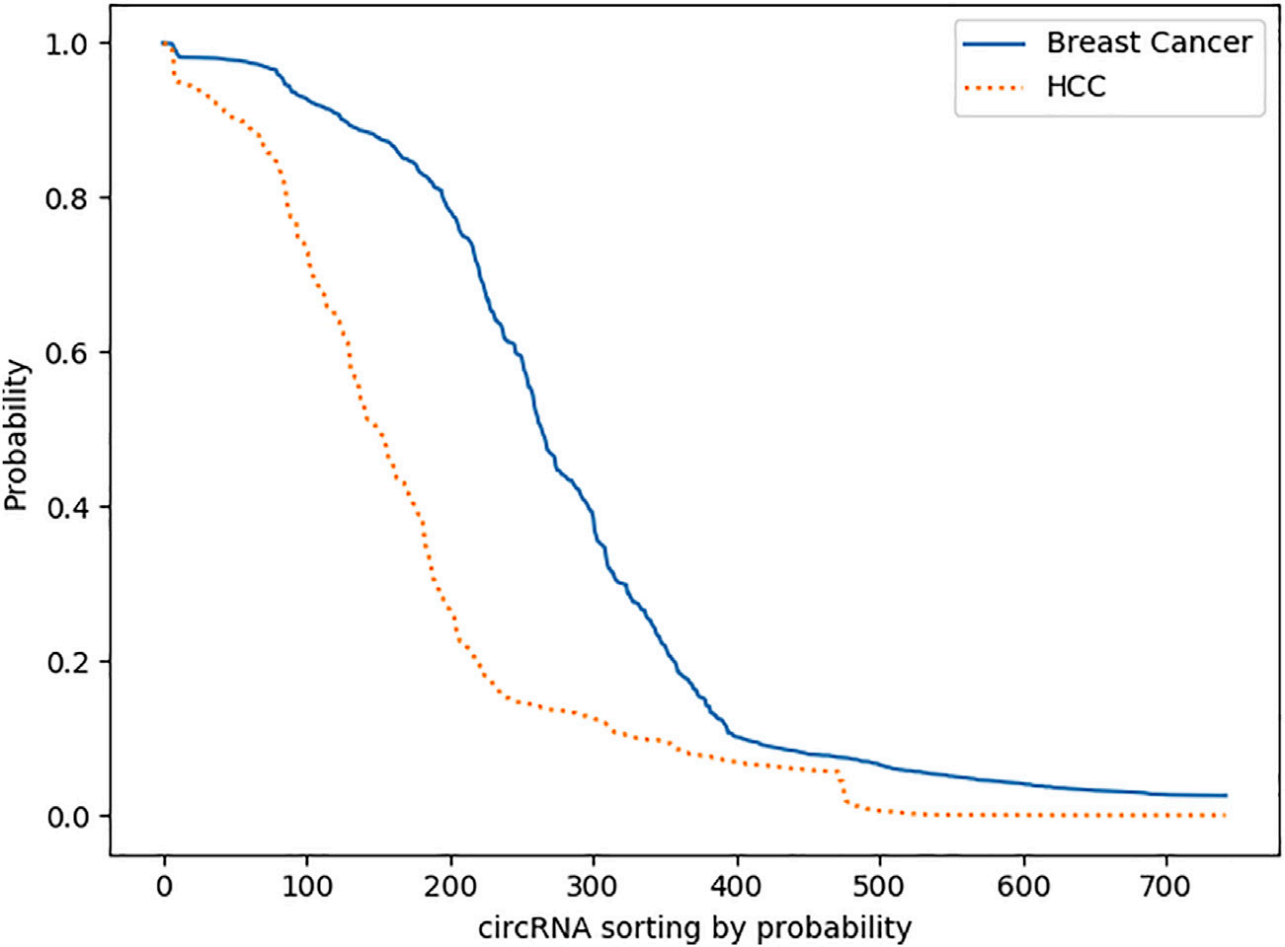
Comparison of predicted probabilities. Both curves decrease from 0.8 to 0.1 significantly, while the curve of Breast Cancer is above the curve of HCC.

We plotted all the predicted results of the two diseases for detailed analysis. Figure 5 shows a significant decrease in both curves from 0.8 to 0.1, which indicates that the model has a strong reliability for each predicted result. Otherwise, the predicted probabilities would be distributed more around 0.5. In addition, a total of 286 data are greater than 0.8, while most of them are less than 0.2; presumably, most circRNA are not associated with breast cancer and HCC, which is in line with the reality.

### 3.4 Prediction and Analysis of Six Diseases

It is worth noting that there is a difference in the prediction performance between breast cancer and HCC. In order to analyze whether it is caused by the difference of data volume in the CircR2Disease dataset, we selected four more diseases, predicted them by using SAAED, and plotted the results in box plots.

**FIGURE 6:**
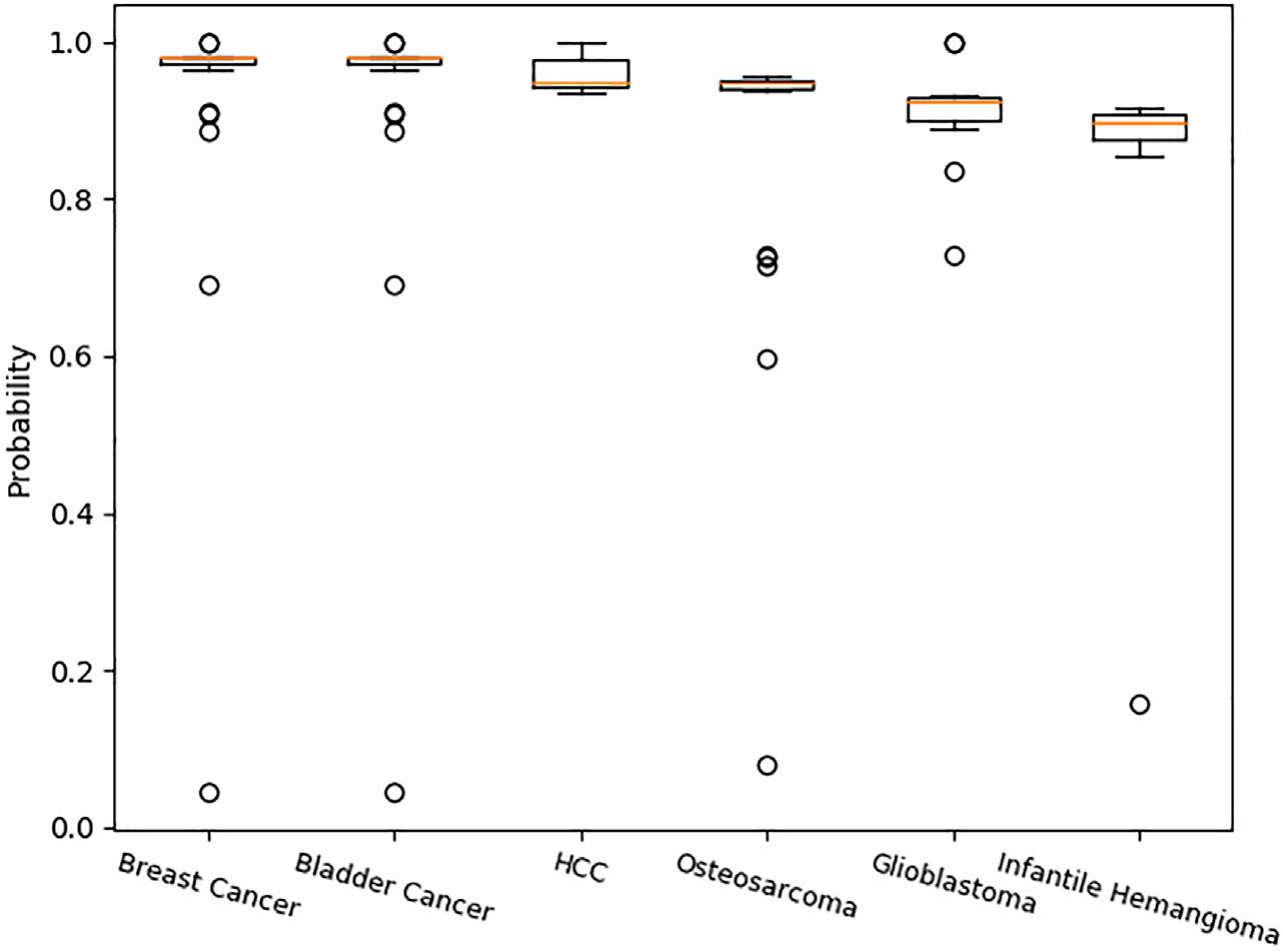
Probability distribution of specific associations in CircR2Disease predicted by SAAED. Figure 6 clearly shows that most probability data are distributed between 0.92 and 1 (except for infantile hemangioma, the median probability distribution is 0.8979), which indicates that SAAED can effectively identify most associations in CircR2Disease. The more the data are, the closer the predicted probability is to 1.

Table 6 shows the amount of related circRNA with diseases in CircR2Disease. The amount of related circRNA with breast cancer is the largest. The amount of related circRNA with infantile hemangioma is the smallest. Table 7 shows the median probability of the related circRNA with the diseases predicted by SAAED. The box plots in Figure 6 clearly show that most probability data are distributed between 0.92 and 1 (except for infantile hemangioma, whose median probability distribution is 0.8979), which indicates that SAAED can effectively identify most associations in CircR2Disease. Meanwhile, the median of each box plot is a common measure used in data centers, which indicates that the more the training data are, the closer the probability distribution is to 1. Therefore, collecting more training data can significantly improve model performance.

**TABLE 6:** Selected circRNA and their amount in CircR2Disease.

Disease	Breast Cancer	Bladder Cancer	HCC	Osteosarcoma	Glioblastoma	Infantile Hemangioma
Amount	58	31	30	22	16	12

**TABLE 7:** Median of the predicted probability.

Disease	Breast Cancer	Bladder Cancer	HCC	Osteosarcoma	Glioblastoma	Infantile Hemangioma
Median	0.9799	0.9412	0.9255	0.9483	0.9235	0.8979

**FIGURE 7:**
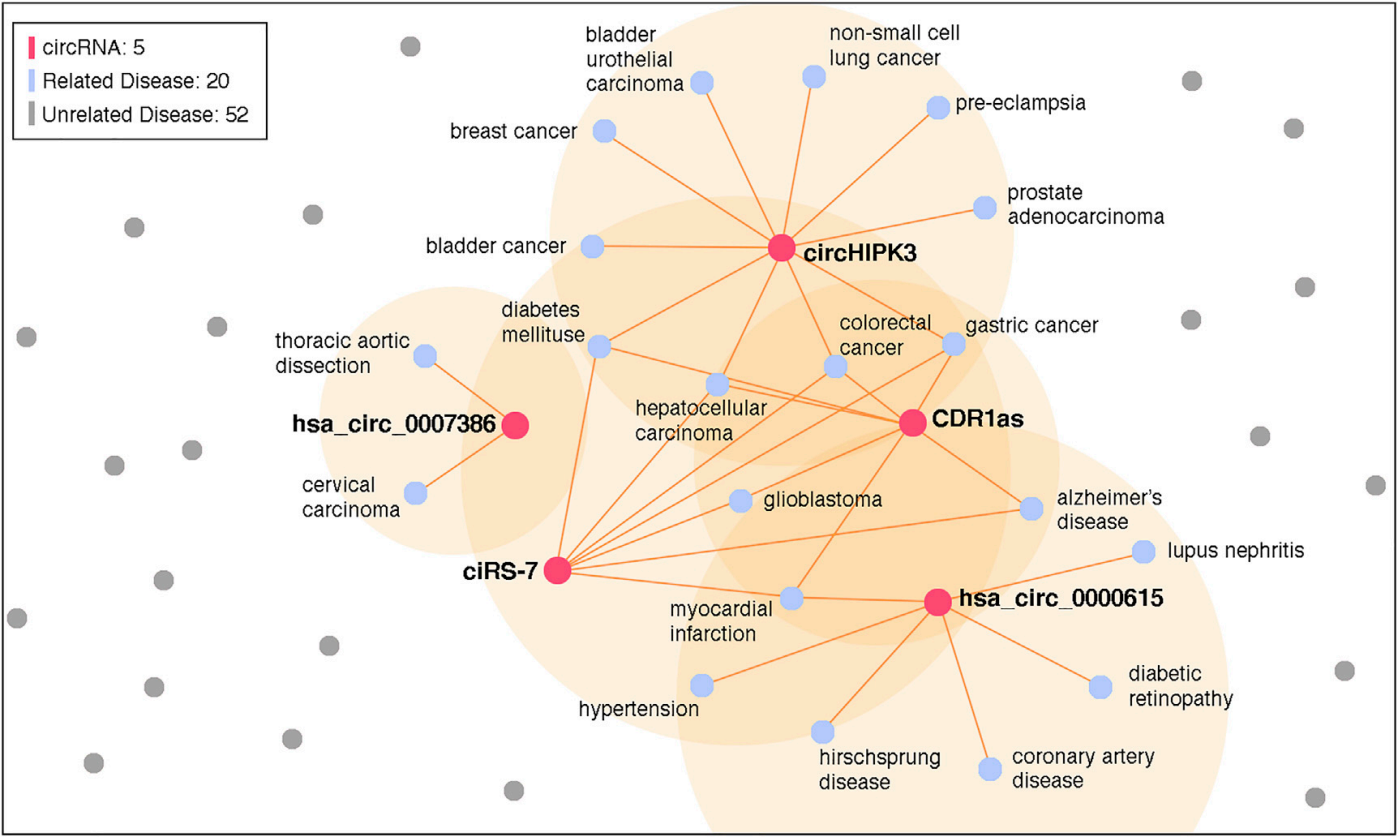
Knowledge graph of the selected circRNA associations. A total of 5 circRNA and 20 related diseases is identified in the graph.

In addition, it is worth noting that the top 10 candidate circRNAs of both breast cancer and HCC are the same. We tried to analyze more diseases and found that most predicted results have similar top 10 candidate circRNAs. We selected the top 5 candidate circRNAs, i.e., hsa_circ_0000615, CDR1as, ciRS-7, hsa_circ_0007386, and circHIPK3 (cZNF609 is an alias of hsa_circ_0000615), and counted their associated diseases in CircR2Disease. The result is visualized in Figure 7. There are 20 associated diseases, which account for 28.6% of all diseases. However, most circRNAs in CircR2Disease are associated with only one disease. We believe that such imbalance of data introduces bias in the model.

## 4 Conclusion

In this study, we proposed a method called SAAED to calculate embedding vectors of circRNA and diseases to predict associations. SAAED consists of ERN and the Pseudo-Siamese network, and ERN is an effective model to calculate entity embedding vectors. The innovative combination of embedding and deep learning can obtain biological association information without adding algorithm complexity. Experimental results show that the model outperforms other state-of-the-art models and can effectively identify circRNA–disease associations. In addition, SAAED can be used for association analysis between any entities. It provides a widely tried path for biological information mining.

It is worth mentioning the limitations of SAAED. First, the reliability of dataset may affect the semantic expression of embedding vector. For example, the imbalance of data in CircR2Disease leads to a similar top 10 associated circRNA of different diseases. Fusing multi-source data and mitigating the bias from different datasets are essential for the generalization and prediction for circRNA–disease association analysis. Second, the data diversity and inconsistency in different datasets are a challenge for data fusing and embedding. We can alleviate this problem by tedious preprocessing, while we believe that the introduction of knowledge graph network is a more effective way to improve the quality of embedding vector and introduce more information.

## Data Availability

The original contributions presented in the study are included in the article/Supplementary Material. Further inquiries can be directed to the corresponding author.
